# Efficacy of Treatments in Reducing Inflammatory Lesion Count in Rosacea: A Systematic Review

**DOI:** 10.1177/12034754241253195

**Published:** 2024-05-28

**Authors:** Ryan S. Q. Geng, Siddhartha Sood, Nicholas Hua, Jennifer Chen, Ronald G. Sibbald, Cathryn Sibbald

**Affiliations:** 1 Temerty School of Medicine, University of Toronto, Toronto, ON, Canada; 2 Dalla Lana School of Public Health and Division of Dermatology, Department of Medicine, University of Toronto, Toronto, ON, Canada; 3 Division of Pediatric Dermatology, The Hospital for Sick Children, University of Toronto, Toronto, ON, Canada

**Keywords:** rosacea, inflammatory lesions, demodex

## Abstract

**Introduction:**

Rosacea is a chronic inflammatory skin condition affecting approximately 5.5% of the global population. Patients present heterogeneously with a mix of features in the central facial region, of which papules and pustules are considered to be a major feature. The identification of effective treatments for reducing inflammatory lesions in rosacea can alleviate the psychosocial burden that many rosacea patients experience, including reduced self-esteem, anxiety, and social withdrawal. The objective of this systematic review is to determine the effectiveness of topical and systemic therapies in reducing lesion count in rosacea patients.

**Methods/Results:**

Medline, Embase, and Cochrane CENTRAL databases were searched, resulting in the inclusion of 43 clinical trials reporting on a total of 18,347 rosacea patients. The most well-studied treatments include ivermectin, metronidazole, azelaic acid, minocycline, and doxycycline. Oral isotretinoin was the most effective treatment in reducing inflammatory lesions and may be recommended for severe recalcitrant cases of rosacea.

**Conclusions:**

Several topical and systemic therapies have demonstrated efficacy in reducing inflammatory lesion count in rosacea patients, with mechanisms of action centred around suppressing inflammation and killing *Demodex folliculorum* mites. Additional research is required to determine effective combination therapies in rosacea.

## Introduction

Rosacea is a chronic inflammatory condition of the skin that affects approximately 5.5% of the global population, with a predilection toward women.^1-3^ Rosacea presents heterogeneously with a mix of features including persistent erythema, phymatous changes, flushing, telangiectasia, papules and pustules, and ocular manifestations.^4^ With the features of rosacea primarily affecting the central facial region, rosacea is associated with substantial psychosocial impact, with many rosacea patients experiencing reduced self-esteem, anxiety, and social withdrawal.^5^

While the pathogenesis of rosacea remains unclear, it is likely multifactorial, involving dysregulation of the innate immune system and the neurovascular system. Several factors including high levels of cathelicidin, activation of inflammasomes, and T_h_1/T_h_17 skewed response are believed to be involved in the pathogenesis of rosacea.^6^ For the formation of inflammatory lesions (ILs; papules and pustules) in rosacea, *Demodex folliculorum* mites in particular have been implicated. The role of *D. folliculorum* in rosacea pathogenesis is supported by the observation that 98.6% of rosacea patients presenting with ILs is found to have high densities of *D. folliculorum*.^7^ *D. folliculorum* mites inhabit hair follicles and have the ability to evade immune responses to establish a persistent presence. However, at higher densities, their inherent immunogenicity may overwhelm the immunosuppressive mechanisms, triggering inflammation and the appearance of ILs.^8^

Several topical and systemic treatment options are available with varying degrees of efficacy in different phenotypes. Given the psychosocial impact of the clinical features of rosacea have on patients, it is important to identify treatments that are effective in alleviating such features. The objective of this systematic review is to determine the effectiveness of topical and systemic therapies in reducing IL count in rosacea patients.

## Methods

### Literature Search

**Figure 1.**
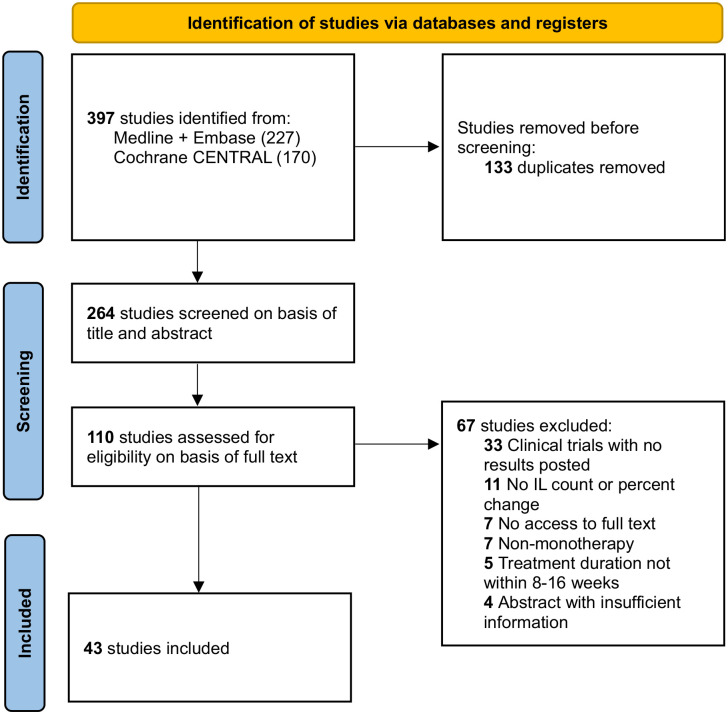
Preferred Reporting Items for Systematic Reviews and Meta-Analyses (PRISMA) flow diagram.

The literature search for this systematic review (CRD42023468649) was performed according to the Preferred Reporting Items for Systematic Reviews and Meta-Analyses (PRISMA) guidelines.^9^ Medline, Embase, and Cochrane CENTRAL were searched from database conception to September 2023, with no exclusions on language. Databases were search with a combination of search terms including rosacea, papules, pustules, and ILs. The full-search strategy is provided in the Supplemental Materials. The removal of 133 duplicates resulted in a total of 264 studies for screening. A PRISMA flow diagram detailing the review process is provided (Figure 1).

### Inclusion and Exclusion Criteria

All clinical trials reporting on the change in IL count posttreatment with a topical or systemic monotherapy with a treatment duration of 8 to 16 weeks were included. Studies that reported the change in ILs by categorical grading scales were excluded.

### Data Extraction and Analysis

Articles were initially screened on basis of title and abstract, followed by full-text screening to determine final eligibility for inclusion. Relevant article references were also assessed for eligibility. NH and JC individually performed data extraction, with disagreements mediated by a third author. The change in IL count data was analyzed by calculating mean percent change in IL count and standard deviations weighted by sample size for each treatment. For studies that report on multiple time points, only the longest time point was included for analysis. The National Institute of Health quality assessment tool was used to assess methodological quality, which provides quality ratings of “good,” “fair,” or “poor.” Only reports with a “good” rating were included.

## Results

**Figure 2.**
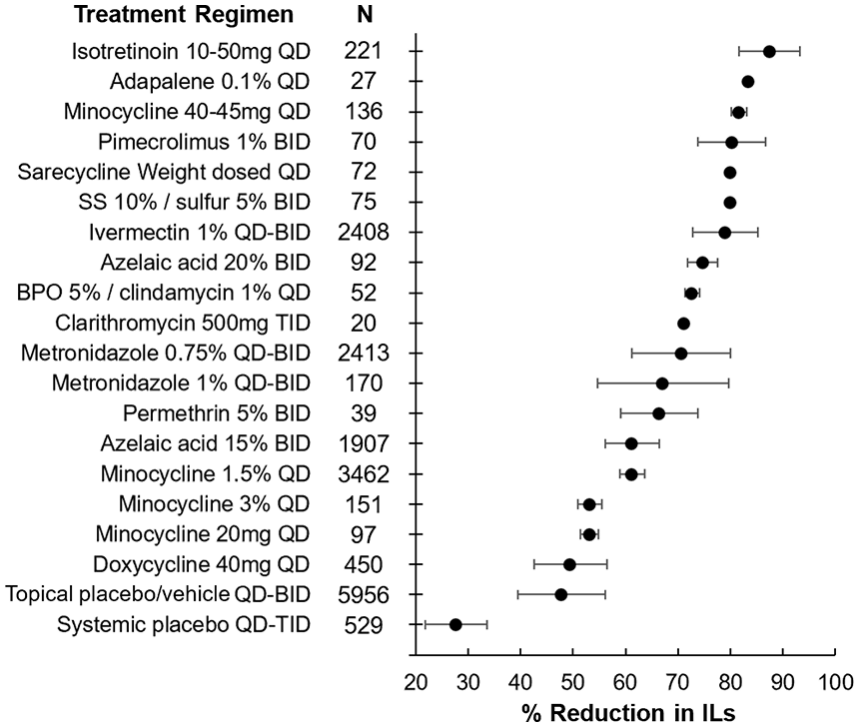
Efficacy of treatment regimens in reducing IL count in rosacea patients. The mean percent reduction in ILs is represented by the bullet for each treatment. Bars represent standard deviations. BPO, benzoyl peroxide; IL, inflammatory lesion; SS, sodium sulfacetamide.

A total of 264 unique articles were identified with the outlined search strategy (Figure 1). Of the 43 articles included, all were randomized controlled trials (RCTs) with the exception of 3 uncontrolled trials, reporting on 18,347 rosacea patients (Supplemental Table 1). A table summarizing the efficacy of treatments in reducing IL counts is provided (Table 1). A diagram ranking the treatments by percent posttreatment IL reduction is also provided (Figure 2).

**Table 1.** Efficacy of Treatment Regimens in Reducing IL Count in Rosacea Patients.

Treatment regimen	N studies	N patients	Mean % reduction in ILs	Fold reduction over placebo
Topical therapies				
Adapalene 0.1% QD	1	27	83.5	1.75
Pimecrolimus 1% BID	3	70	80.3	1.68
SS 10%/sulfur 5% BID	1	75	80	1.67
Ivermectin 1% QD-BID	6	2408	79	1.65
Azelaic acid 20% BID	2	92	74.7	1.56
BPO 5%/clindamycin 1% QD	1	52	72.7	1.52
Metronidazole 0.75% QD-BID	13	2413	70.6	1.48
Metronidazole 1% QD-BID	3	170	67.2	1.41
Permethrin 5% BID	2	39	66.5	1.39
Azelaic acid 15% BID	5	1907	61.3	1.28
Minocycline 1.5% QD	7	3462	61.3	1.28
Minocycline 3% QD	2	151	53.2	1.11
Placebo/vehicle QD-BID	23	5956	47.8	—
Systemic therapies				
Isotretinoin 10-50 mg QD	3	221	87.5	3.17
Minocycline 40-45 mg QD	3	136	81.6	2.96
Sarecycline weight-dosed QD	1	72	80	2.90
Clarithromycin 500 mg TID	1	20	71.2	2.58
Minocycline 20 mg QD	2	97	53.2	1.93
Doxycycline 40 mg QD	4	450	49.5	1.79
Systemic placebo QD-TID	6	529	27.6	—

Abbreviations: BID, twice daily; BPO, benzoyl peroxide; IL, inflammatory lesion; QD, once daily; SS, sodium sulfacetamide; TID, three times daily.

### Adapalene (27 Patients)

Adapalene was reported by 1 study in a 0.1% topical formulation applied once daily.^10^ There was a mean 83.5% reduction in ILs among the 27 patients, representing a 1.75-fold reduction over topical placebo/vehicle.

### Azelaic acid (1999 Patients)

Azelaic acid was reported by 7 studies in either 15% or 20% topical formulations applied twice daily.^11-17^ For the 2 studies reporting on the 20% formulation, there was a mean 74.7% reduction in ILs among the 92 patients, representing a 1.56-fold reduction over topical placebo/vehicle.^11,14^ For the 5 studies reporting on the 15% formulation, there was a mean 61.3% reduction in ILs among the 1907 patients, representing a 1.28-fold reduction over topical placebo/vehicle.^12,13,15-17^

### Benzoyl Peroxide/Clindamycin (52 Patients)

Benzoyl peroxide 5%/clindamycin 1% topical formulation was reported by 1 study.^18^ There was a mean 72.7% reduction in ILs among the 52 patients, representing a 1.52-fold reduction over topical placebo/vehicle.

### Clarithromycin (20 Patients)

Clarithromycin was reported by 1 study at a dose of 500 mg thrice daily.^19^ There was a mean 71.2% reduction in ILs among the 20 patients, representing a 2.58-fold reduction over systemic placebo.

### Doxycycline (450 Patients)

Doxycycline was reported by 4 studies at a dose of 40 mg once daily.^20-23^ There was a mean 49.5% reduction in ILs among the 450 patients, representing a 1.79-fold reduction over systemic placebo.

### Isotretinoin (141 Patients)

Isotretinoin was reported by 3 studies at doses ranging between of 10 and 50 mg once daily, with doses studied including mean 33.3 mg/day (82.1% IL reduction), 20 mg/day (90.9% IL reduction), and mean 15.6 mg/day (92% IL reduction).^24-26^ There was a mean 87.5% reduction in ILs among the 221 patients, representing a 3.17-fold reduction over systemic placebo.

### Ivermectin (2408 Patients)

Ivermectin was reported by 6 studies in a 1% formulation applied once or twice daily.^27-32^ There was a mean 79% reduction in ILs among the 2408 patients, representing a 1.65-fold reduction over topical placebo/vehicle.

### Metronidazole (2583 Patients)

Metronidazole was reported by 16 studies in either 0.75% or 1% formulations applied once or twice daily.^10,13,14,28,31-42^ For the 13 studies reporting on the 0.75% formulation, there was a mean 70.6% reduction in ILs among the 2413 patients, representing a 1.48-fold reduction over topical placebo/vehicle.^10,13,14,28,31-33,35,36,38,39,41,42^ For the 3 studies reporting on the 1% formulation, there was a mean 67.2% reduction in ILs among the 170 patients, representing a 1.41-fold reduction over topical placebo/vehicle.^34,36,37,40^

### Minocycline (3846 Patients)

Minocycline was reported by 10 studies in either 1.5% or 3% topical formulations applied once daily or dosed between 20 and 45 mg once daily.^22,23,43-50^

For the 7 studies reporting on the 1.5% formulation, there was a mean 45.9% decrease in ILs among the 3462 patients, representing a 1.28-fold reduction over topical placebo.^43-45,47-50^ For the 2 studies reporting on the 3% formulation, there was a mean 53.2% reduction in ILs among the 151 patients, representing a 1.11-fold reduction over topical placebo/vehicle.^47,50^

For the 2 studies reporting on a 20 mg once-daily dose, there was a mean 53.2% reduction in ILs among the 97 patients, representing a 1.93-fold reduction over systemic placebo.^22,23^ For the 3 studies reporting on a 40 to 45 mg once-daily dose, there was a mean 81.6% reduction in ILs among the 136 patients, representing a 2.96-fold reduction over systemic placebo.^22,23,46^

### Permethrin (39 Patients)

Permethrin was reported by 2 studies in a 5% formulation applied twice daily.^14,38^ There was a mean 66.5% reduction in ILs among the 39 patients, representing a 1.39-fold reduction over topical placebo/vehicle.

### Pimecrolimus (70 Patients)

Pimecrolimus was reported by 3 studies in a 1% formulation applied twice daily.^37,51,52^ There was a mean 80.3% reduction in ILs among the 70 patients, representing a 1.68-fold reduction over topical placebo/vehicle.

### Sarecycline (72 Patients)

Sarecycline was reported by 1 study at a once-daily weight-based dose according to the label.^48^ There was a mean 80% reduction in ILs among the 72 patients, representing a 2.90-fold reduction over systemic placebo.

### Sodium Sulfacetamide/Sulphur (75 Patients)

Sodium sulfacetamide 10%/sulphur 5% topical formulation was reported by 1 study.^41^ There was a mean 80% reduction in ILs among the 75 patients, representing a 1.67-fold reduction over topical placebo/vehicle.

## Discussion

This review included 43 articles reporting on a total of 18,347 rosacea patients, assessing the efficacy of 13 different topical or systemic treatments in reducing IL count. The quality of evidence in this review overall was high, with all included articles being RCTs, with the exception of 3 uncontrolled trials.

Systemic treatments generally achieved greater fold reductions in IL count over placebo compared to topical treatments. However, this is largely due to the high difference in percent IL reduction between topical placebo/vehicle and systemic placebo, 47.8% and 27.6%, respectively (Table 1). Studies assessing topical therapies often use vehicle formulations as the comparator rather than placebo. Unlike placebo, vehicle formulations function to enhance absorption and activity of drugs, and are not merely inert excipients. Rather, they often contain components that aid in skin barrier restoration and moisturization, which can soothe inflammation.^53^ These properties cannot be provided by a systemic placebo.

Among the systemic treatments, isotretinoin in a 10 to 50 mg daily dosage achieved the greatest percent reductions in IL count. Isotretinoin is most commonly used in treating severe forms of acne vulgaris, with its efficacy due to its ability to inhibit comedogenesis, reduce sebum excretion, and pilosebaceous unit size.^54^ While ILs in rosacea do not arise from comedones, the reduction in sebum excretion and pilosebaceous unit size may be contributory to its efficacy in rosacea. *D. folliculorum* are believed to play a large role in formation of ILs in rosacea. These mites inhabit hair follicles and feed on sebum and skin cells.^55^ While isotretinoin does not boast acaricidal properties, it appears to indirectly inhibit mite proliferation. The atrophy of pilosebaceous units and reduced sebum secretion induced by isotretinoin therapy may reduce the hospitability of the host skin and inhibit mite proliferation. Thus, isotretinoin is able to modify the conditions in the skin that promote demodex mite proliferation, which could explain why it is more effective than agents that simply kill the mite, such as ivermectin. In an ex vivo study, isotretinoin was found to reduce demodex mite density by 63.4%.^56^ Isotretinoin has been demonstrated to be effective in treating rosacea, particularly in cases of severe recalcitrant rosacea with papules and pustules.^26^ Isotretinoin can also inhibit neutrophil and monocyte chemotaxis, providing an anti-inflammatory effects that may also be beneficial in rosacea.^57^

Doxycycline was the most commonly studied systemic therapy, being used at a subantimicrobial anti-inflammatory dosage of 40 mg daily. Subantimicrobial dosages are preferred to prevent changes to gut microbiota and reduce risk of gastrointestinal adverse reactions and development of resistance.^6^ The efficacy of doxycycline in treating rosacea is likely due to its ability to downregulate several factors involved in rosacea pathogenesis including interleukin 1β, tumour necrosis factor α (TNFα), kallikrein-5 (KLK5), cathelicidin, and matrix metalloprotease 9.^58,59^

The most commonly studied topical treatments were ivermectin, metronidazole, azelaic acid, and minocycline. Among these, ivermectin achieved the greatest percent reduction in ILs, which can be explained by its direct acaricidal and anti-inflammatory properties via inhibition of interleukin 1β and TNFα. However, some patients may experience an acute exacerbation of symptoms due to the sudden death of demodex mites.^6^ This transient inflammatory exacerbation is likely due to the release of immunogenic antigens from the dead mites, but may also be linked to the release of *Bacillus oleronius* bacteria that live inside the mites. *B. oleronius* is not a human cutaneous commensal organism and has been shown to trigger an inflammatory response in 73% of rosacea patients.^60^ Thus, targeting *B. oleronius* may also provide therapeutic benefit to rosacea patients. Interestingly, *B. oleronius* is susceptible to several antibiotics commonly used in rosacea, including metronidazole, doxycycline, and minocycline.^60,61^ While metronidazole does not exhibit acaricidal activity toward demodex mites, it has reactive oxygen species scavenging activity, inhibits production of pro-inflammatory cytokines, and exhibits bactericidal activity against *B. oleronius*.^62,63^ The efficacy of azelaic acid is due to its ability to suppress KLK5 and cathelicidin production, which are part of a major inflammatory pathway contributing to rosacea pathogenesis.^64^ Topical minocycline 1.5% foam was comparable to azelaic acid 15% cream, and is believed to owe its efficacy in treating rosacea to its anti-inflammatory properties.^6^

Given that several topical and systemic therapies are effective in treating ILs in rosacea with differing mechanisms of action, combination therapies may be especially effective. Indeed, in 1 trial, patients treated with doxycycline 40 mg and ivermectin 1% cream once daily exhibited a mean 80.3% reduction in IL count compared to 73.6% for those treated with ivermectin monotherapy (*P* = .032). Combination therapy also demonstrated a faster onset of action, with significant differences noted by week 4.^65^

Limitations of our study include a large range of patient sample sizes and heterogenous patient population. Rosacea is a chronic disease, with periods of remission and relapse. The results of this study are only applicable to the reduction of active ILs, and does not reflect remission of disease. Additional research is required to determine the effectiveness of various combination treatments in reducing IL count in rosacea.

## Conclusion

Rosacea is a psychosocially burdensome chronic inflammatory skin condition affecting approximately 5.5% of the global population. A major feature of rosacea is ILs, consisting of papules and pustules. Several topical and systemic therapies have demonstrated efficacy in reducing IL count in rosacea patients, with mechanisms of action centred around suppressing inflammation and killing *D. folliculorum* mites. The most well-studied treatments for ILs in rosacea include ivermectin, metronidazole, azelaic acid, doxycycline, and minocycline. Isotretinoin was found to be the most effective and could be considered for cases of severe recalcitrant rosacea.

## Supplemental Material

sj-docx-1-cms-10.1177_12034754241253195 – Supplemental material for Efficacy of Treatments in Reducing Inflammatory Lesion Count in Rosacea: A Systematic Review Supplemental material, sj-docx-1-cms-10.1177_12034754241253195 for Efficacy of Treatments in Reducing Inflammatory Lesion Count in Rosacea: A Systematic Review by Ryan S. Q. Geng, Siddhartha Sood, Nicholas Hua, Jennifer Chen, Ronald G. Sibbald and Cathryn Sibbald in Journal of Cutaneous Medicine and Surgery

sj-docx-2-cms-10.1177_12034754241253195 – Supplemental material for Efficacy of Treatments in Reducing Inflammatory Lesion Count in Rosacea: A Systematic Review Supplemental material, sj-docx-2-cms-10.1177_12034754241253195 for Efficacy of Treatments in Reducing Inflammatory Lesion Count in Rosacea: A Systematic Review by Ryan S. Q. Geng, Siddhartha Sood, Nicholas Hua, Jennifer Chen, Ronald G. Sibbald and Cathryn Sibbald in Journal of Cutaneous Medicine and Surgery

sj-docx-3-cms-10.1177_12034754241253195 – Supplemental material for Efficacy of Treatments in Reducing Inflammatory Lesion Count in Rosacea: A Systematic Review Supplemental material, sj-docx-3-cms-10.1177_12034754241253195 for Efficacy of Treatments in Reducing Inflammatory Lesion Count in Rosacea: A Systematic Review by Ryan S. Q. Geng, Siddhartha Sood, Nicholas Hua, Jennifer Chen, Ronald G. Sibbald and Cathryn Sibbald in Journal of Cutaneous Medicine and Surgery
