# Supplementary material for: Efficacy of Treatments in Reducing Inflammatory Lesion Count in Rosacea: A Systematic Review
Source: J Cutan Med Surg. 2024 May 28;28(4):352–9. doi: 10.1177/12034754241253195 (PMC11408985; doi:10.1177/12034754241253195)
Supplement: sj-docx-1-cms-10.1177_12034754241253195 – Supplemental material for Efficacy of Treatments in Reducing Inflammatory Lesion Count in Rosacea: A Systematic Review [file sj-docx-1-cms-10.1177_12034754241253195.docx]

**Figure S1.** PRISMA flow diagram

**Identification of studies via databases and registers**

**Identification**

**Screening**

**Included**

**397** studies identified from:

Medline + Embase (227)

Cochrane CENTRAL (170)

Studies removed before screening:

**133** duplicates removed

**264** studies screened on basis of title and abstract

**110** studies assessed for eligibility on basis of full text

**67** studies excluded:

**33** Clinical trials with no results posted

**11** No IL count or percent change

**7** No access to full text

**7** Non-monotherapy

**5** Treatment duration not within 8-16 weeks

**4** Abstract with insufficient information

**43** studies included
