# Supplementary material for: Efficacy of Treatments in Reducing Inflammatory Lesion Count in Rosacea: A Systematic Review
Source: J Cutan Med Surg. 2024 May 28;28(4):352–9. doi: 10.1177/12034754241253195 (PMC11408985; doi:10.1177/12034754241253195)
Supplement: sj-docx-2-cms-10.1177_12034754241253195 – Supplemental material for Efficacy of Treatments in Reducing Inflammatory Lesion Count in Rosacea: A Systematic Review [file sj-docx-2-cms-10.1177_12034754241253195.docx]

**Supplementary**

**Search strategy**

**Medline and Embase**

1 rosacea.ab,ti.

2 papule.ab,ti.

3 pustule.ab,ti.

4 "inflammatory lesion".ab,ti.

5 2 or 3 or 4

6 1 and 5

**Cochrane central**

(Rosacea):ti,ab,kw AND (papule OR pustule OR inflammatory lesion):ti,ab,kw

**Supplemental Table 1.** Studies included in this review showing treatment, sample size, inflammatory lesion count and time-point data.

| **Article** | **Treatment regimen** | **N patients** | **Pre-treatment IL count (SD)** | **Post-treatment IL count (SD)** | **% Decrease** | **Time point (weeks)** |
| --- | --- | --- | --- | --- | --- | --- |
| Altinyazar 2005^1^ | Adapalene 0.1% QD | 27 | 12.1 (NR) | 2 (NR) | 83.5 | 12 |
|  | Metronidazole 0.75% BID | 23 | 12.04 (NR) | 2.72 (NR) | 77.4 | 12 |
| Aronson 1987^2^ | Metronidazole 0.75% NR | 46 | NR | NR | 51.0 | 9 |
| Bamford 1999^3^ | Clarithromycin 500mg TID | 20 | 21.5 (14.8) | 6.2 (8.3) | 71.2 | 8.5 |
|  | Placebo TID | 22 | 22 (18.7) | 12.6 (19.3) | 42.7 | 8.5 |
| Bitar 1990^4^ | Metronidazole 1% BID | 50 | 11.3 (0.81) | 4.5 (0.76) | 60.2 | 8 |
|  | Placebo BID | 50 | 13.2 (0.92) | 10.2 (1.48) | 22.7 | 8 |
| Bjerke 1999^5^ | Azelaic acid 20% BID | 76 | 30.8 | 8.3 | 73.4 | 12 |
|  | Vehicle BID | 38 | 31.7 | 15.3 | 50.6 | 12 |
| Bleicher 1987^6^ | Metronidazole 0.75% BID | 34 | NR | NR | 48.2 | 12 |
|  | Vehicle BID | 34 | NR | NR | 23.8 | 12 |
| Breneman 2004^7^ | Benzoyl peroxide 5% / clindamycin 1% QD | 26 | 17.7 (9.7) | NR | 74.1 | 12 |
|  | Benzoyl peroxide 5% / clindamycin 1% QD | 26 | 17.7 (9.7) | NR | 71.3 | 12 |
|  | Vehicle QD | 26 | 19.3 (11.4) | NR | 36.7 | 12 |
|  | Vehicle QD | 26 | 19.3 (11.4) | NR | 19.3 | 12 |
| Dahl 2001^8^ | Metronidazole 0.75% QD | 35 | 19 | 17 | 10.5 | 12 |
|  | Metronidazole 0.75% QD | 35 | 19 | 9 | 52.6 | 12 |
|  | Metronidazole 1% QD | 35 | 25 | 10 | 60.0 | 12 |
| DelRosso 2007^9^ | Doxycycline 40mg QD | 127 | 19.5 (8.8) | 7.7 | 60.5 | 16 |
|  | Doxycycline 40mg QD | 142 | 20.5 (11.7) | 11 | 46.3 | 16 |
|  | Placebo QD | 124 | 20.3 (10.4) | 14.4 | 29.1 | 16 |
|  | Placebo QD | 144 | 21.2 (12.5) | 16.9 | 20.3 | 16 |
| DelRosso 2021^10^ | Minocycline 1.5% QD | 1009 | 29.2 (12.48) | 11.2 | 61.6 | 12 |
|  | Vehicle QD | 513 | 29.6 (12.57) | 14.7 | 50.3 | 12 |
| DiNardo 2016^11^ | Doxycycline 40mg QD | 84 | NR | NR | 44.6 | 12 |
|  | Placebo QD | 86 | NR | NR | 25.2 | 12 |
| Draelos 2015^12^ | Azelaic acid 15% BID | 484 | NR | NR | 61.6 | 12 |
|  | Vehicle BID | 477 | NR | NR | 50.9 | 12 |
| Elewski 2003^13^ | Azelaic acid 15% BID | 124 | 18.1 | 4.5 | 75.7 | 15 |
|  | Metronidazole 0.75% BID | 127 | 19.4 | 7.6 | 61.3 | 15 |
| EUCTR2010-018319-13-HU 2010^14^ | Ivermectin 1% QD | 104 | 35.2 (17.0) | 8.6 (11.7) | 74.5 | 12 |
|  | Vehicle QD | 106 | 40.0 (28.1) | 17.2 (23.5) | 59.3 | 12 |
| Foley 2016^15^ | Ivermectin 1% BID | 478 | NR | NR | 83.0 | 16 |
|  | Metronidazole 0.75% BID | 484 | NR | NR | 73.7 | 16 |
| Gold 2014^16^ | Ivermectin 1% QD | 414 | NR | NR | 76.0 | 12 |
|  | Ivermectin 1% QD | 429 | NR | NR | 75.0 | 12 |
|  | Vehicle QD | 210 | NR | NR | 50.0 | 12 |
|  | Vehicle QD | 208 | NR | NR | 50.0 | 12 |
| Gold 2020^17^ | Minocycline 1.5% QD | 437 | NR | NR | 64.0 | 12 |
|  | Minocycline 1.5% QD | 479 | NR | NR | 61.0 | 12 |
|  | Vehicle QD | 232 | NR | NR | 57.0 | 12 |
|  | Vehicle QD | 239 | NR | NR | 50.0 | 12 |
| Gold 2020^18^ | Minocycline 1.5% QD | 332 | 28.8 (12.63) | 10.9 | 62.4 | 12 |
|  | Vehicle QD | 172 | 28.7 (11.93) | 15.9 | 46.4 | 12 |
| *Hoting 1986^19^ | Isotretinoin 10-50mg QD | 80 | 53 | 9.5 | 82.1 | 12 |
| Jackson 2013^20^ | Minocycline 45mg QD | 30 | 15 (7) | 3 (5) | 80.0 | 16 |
| Koca 2010^21^ | Metronidazole 1% BID | 24 | 16.0 (4.6) | 0.6 (1.5) | 97.3 | 12 |
|  | Pimecrolimus 1% BID | 25 | 26.0 (11.7) | 3.7 (6.8) | 88.9 | 12 |
| Koçak 2002^22^ | Metronidazole 0.75% BID | 20 | 12.9 (8.23) | 5.30 (4.18) | 58.9 | 8 |
|  | Permethrin 5% BID | 23 | 8.34 (8.47) | 2.29 (2.54) | 72.5 | 8 |
|  | Placebo BID | 20 | 7.45 (5.30) | 7.50 (4.90) | -0.7 | 8 |
| Lee 2008^23^ | Pimecrolimus 1% BID | 15 | 18.0 (24.1) | 4.3 (8.0) | 76.1 | 8 |
| Maddin 1999^24^ | Pimecrolimus 1% BID | 15 | 19.5 (27.9) | 5.0 (9.9) | 74.4 | 8 |
|  | Pimecrolimus 1% BID | 15 | 18 (24.1) | 4.3 (8) | 76.1 | 8 |
| Miyachi 2021^25^ | Metronidazole 0.75% BID | 65 | NR | NR | 76.4 | 12 |
|  | Vehicle BID | 65 | NR | NR | 27.5 | 12 |
| Mostafa 2009^26^ | Azelaic acid 20% BID | 16 | 4.2 (2.7) | 0.8 (0.48) | 81.0 | 15 |
|  | Metronidazole 0.75% BID | 16 | 5.4 (3.3) | 1.7 (0.5) | 68.5 | 15 |
|  | Permethrin 5% BID | 16 | 4.5 (3.7) | 1.9 (0.6) | 57.8 | 15 |
| Mrowietz 2018^27^ | Minocycline 1.5% BID | 79 | 34.5 | 13.4 | 61.4 | 12 |
|  | Minocycline 3% BID | 75 | 34.1 | 14.2 | 55.5 | 12 |
|  | Vehicle QD | 78 | 30.6 | 22.8 | 29.7 | 12 |
| NCT01555463 2012^28^ | Azelaic acid 15% BID | 483 | 21.7 (9.1) | 8.5 (8.9) | 60.8 | 12 |
|  | Vehicle BID | 478 | 21.2 (8.7) | 10.8 (11.3) | 49.1 | 12 |
| NCT02795117 2016^29^ | Ivermectin 1% QD | 156 | NR | NR | 65.5 | 12 |
|  | Ivermectin 1% QD | 147 | NR | NR | 67.9 | 12 |
|  | Placebo NR | 75 | NR | NR | 55.5 | 12 |
| NCT03340961 2017^30^ | Doxycycline 40mg QD | 48 | 23.8 (7.56) | 13.3 | 44.1 | 16 |
|  | Minocycline 20mg QD | 47 | 24.5 (9.49) | 11.9 | 51.4 | 16 |
|  | Minocycline 40mg QD | 53 | 23.8 (8.69) | 4.6 | 80.7 | 16 |
|  | Placebo QD | 52 | 24 (8.4) | 16.7 | 30.4 | 16 |
| NCT04555525 2020^31^ | Sarecycline weight dosed QD | 72 | NR | NR | 80.0 | 12 |
|  | Minocycline 1.5% QD | 474 | NR | NR | 61.5 | 12 |
|  | Placebo QD | 235 | NR | NR | 50.2 | 12 |
| Sbidian 2016^32^ | Isotretinoin 0.24mg/kg QD | 108 | 17 | NR | 92.0 | 16 |
|  | Placebo QD | 48 | 15 | NR | 36.0 | 16 |
| Schaller 2016^33^ | Ivermectin 1% QD | 478 | NR | NR | 85.1 | 16 |
|  | Metronidazole 0.75% BID | 484 | NR | NR | 75.2 | 16 |
| Solomon 2016^34^ | Azelaic acid 15% BID | 483 | 21.7 | NR | 61.6 | 12 |
|  | Vehicle BID | 478 | 21.2 | NR | 50.8 | 12 |
| SteinGold 2019^35^ | Minocycline 1.5% QD | 495 | 28.5 (12.05) | 10.93 | 61.7 | 12 |
|  | Minocycline 1.5% QD | 514 | 30 (12.84) | 11.46 | 61.8 | 12 |
|  | Vehicle QD | 256 | 29 (12.13) | 13.35 | 54.0 | 12 |
|  | Vehicle QD | 257 | 30.2 (12.99) | 15.32 | 49.3 | 12 |
| Taieb 2015^36^ | Ivermectin 1% QD | 478 | 32.87 (13.95) | NR | 83.0 | 16 |
|  | Metronidazole 0.75% BID | 484 | 32.07 (12.75) | NR | 73.7 | 16 |
| Tan 2002^37^ | Metronidazole 1% BID | 61 | 19.6 (2.7) | NR | 65.1 | 12 |
|  | Placebo BID | 59 | 20.3 (1.8) | NR | 25.4 | 12 |
| Thiboutot 2003^38^ | Azelaic acid 15% BID | 164 | 17.5 | 6.8 | 61.1 | 12 |
|  | Azelaic acid 15% BID | 169 | 17.8 | 8.9 | 50.0 | 12 |
|  | Vehicle BID | 165 | 17.6 | 10.5 | 40.3 | 12 |
|  | Vehicle BID | 166 | 18.5 | 12.1 | 34.6 | 12 |
| Torok 2005^39^ | Metronidazole 0.75% BID | 77 | 17 (1) | NR | 72.0 | 12 |
|  | Sodium sulfacetamide 10% / sulfur 5% BID | 75 | 18 (1) | NR | 80.0 | 12 |
| Tsianakas 2021^40^ | Doxycycline 40mg QD | 49 | 24 | 13.5 | 43.8 | 16 |
|  | Minocycline 20mg QD | 50 | 23 | 10.4 | 54.8 | 16 |
|  | Minocycline 40mg QD | 53 | 23 | 3.8 | 83.5 | 16 |
|  | Placebo QD | 53 | 23 | 15.7 | 31.7 | 16 |
| *Uslu 2012^41^ | Isotretinoin 20mg QD | 25 | 11 | 1 | 90.9 | 16 |
| Webster 2020^42^ | Minocycline 1% QD | 80 | 24.6 (7.4) | 13.3 | 45.9 | 16 |
|  | Minocycline 3% QD | 76 | 25.1 (7.9) | 12.3 | 51.0 | 16 |
|  | Vehicle QD | 63 | 24.3 (8.2) | 16.3 | 32.9 | 16 |
| *WolfJr 2007^43^ | Metronidazole 0.75% BID | 446 | 15.6 (3.67) | 4.5 (2.93) | 71.2 | 12 |

All studies included in this review were RCTs, except for three uncontrolled trials denoted by an asterisk.

**References**

1. Altinyazar HC, Koca R, Tekin NS, Eştürk E. Adapalene vs. metronidazole gel for the treatment of rosacea. 2005;44(3):252‐5.

2. Aronson IK, Rumsfield JA, West DP, Alexander J, Fischer JH, Paloucek FP. Evaluation of topical metronidazole gel in acne rosacea. 1987;21(4):346‐51.

3. Bamford JT, Tilden RL, Blankush JL, Gangeness DE. Effect of treatment of Helicobacter pylori infection on rosacea. 1999;135(6):659‐63.

4. Bitar A, Bourgouin J, Dore N, Dubuc R, Giroux JM, Landry M, et al. A double-blind randomised study of metronidazole (Flagyl®) 1% cream in the treatment of acne rosacea. A placebo-controlled study. 1990;2(4):242‐8.

5. Bjerke R, Fyrand O, Graupe K. Double-blind comparison of azelaic acid 20% cream and its vehicle in treatment of papulo-pustular rosacea. 1999;79(6):456‐9.

6. Bleicher PA, Charles JH, Sober AJ. Topical metronidazole therapy for rosacea. 1987;123(5):609‐14.

7. Breneman D, Savin R, VandePol C, Vamvakias G, Levy S, Leyden J. Double-blind, randomized, vehicle-controlled clinical trial of once-daily benzoyl peroxide/clindamycin topical gel in the treatment of patients with moderate to severe rosacea. 2004;43(5):381‐7.

8. Dahl MV, Jarratt M, Kaplan D, Tuley MR, Baker MD. Once-daily topical metronidazole cream formulations in the treatment of the papules and pustules of rosacea. 2001;45(5):723‐30.

9. Del Rosso JQ, Webster GF, Jackson M, Rendon M, Rich P, Torok H, et al. Two randomized phase III clinical trials evaluating anti-inflammatory dose doxycycline (40-mg doxycycline, USP capsules) administered once daily for treatment of rosacea. Journal of the American Academy of Dermatology. 2007;56(5):791-802.

10. Del Rosso JQ, Stein Gold L, Kircik L, Bhatia ND, Sadick N, Zirwas M, et al. Integrated safety and efficacy analysis of FMX103 1.5% topical minocycline foam for the treatment of moderate-to-severe papulopustular rosacea: results from two Phase III studies. 2021;14(5 SUPPL 1):S30.

11. Di Nardo A, Holmes AD, Muto Y, Huang EY, Preston N, Winkelman WJ, et al. Improved clinical outcome and biomarkers in adults with papulopustular rosacea treated with doxycycline modified-release capsules in a randomized trial. 2016;74(6):1086‐92.

12. Draelos ZD, Elewski BE, Harper JC, Sand M, Staedtler G, Nkulikiyinka R, et al. Randomized, phase III, double-blind, vehicle-controlled clinical trial to evaluate the safety and efficacy of 12 weeks of twice-daily azelaic acid foam, 15% in papulopustular rosacea. Journal of the American Academy of Dermatology. 2015;72(5 SUPPL. 1):AB59.

13. Elewski BE, Fleischer Jr AB, Pariser DM. A Comparison of 15% Azelaic Acid Gel and 0.75% Metronidazole Gel in the Topical Treatment of Papulopustular Rosacea: Results of a Randomized Trial. Arch Dermatol. 2003;139(11):1444-50.

14. Euctr HU. A DOUBLE BLIND, VEHICLE CONTROLLED, PARALLEL GROUP STUDY ASSESSING THE ACTIVITY OF CD5024 1% CREAM IN SUBJECTS WITH PAPULOPUSTULAR ROSACEA OVER 12 WEEKS TREATMENT. 2010.

15. Foley P, Taieb A, Ruzicka T, Peirone MH, Jacovella J. Comparative efficacy and safety of ivermectin 1% cream and metronidazole 0.75% cream in the novel treatment of papulopustular rosacea: The ATTRACT (assessment of a topical treatment in rosacea - activity, compliance, tolerability) study. Australas J Dermatol. 2016;57(SUPPL. 1):33.

16. Gold LS, Kircik L, Fowler J, Tan J, Draelos Z, Fleischer A, et al. Efficacy and safety of ivermectin 1% cream in treatment of papulopustular rosacea: Results of two randomized, double-blind, vehicle-controlled pivotal studies. Journal of Drugs in Dermatology. 2014;13(3):316-23.

17. Gold LS, Del Rosso JQ, Kircik L, Bhatia ND, Hooper D, Nahm WK, et al. Minocycline 1.5% foam for the topical treatment of moderate to severe papulopustular rosacea: results of 2 phase 3, randomized, clinical trials. 2020;82(5):1166‐73.

18. Gold LS, Del Rosso JQ, Kircik L, Bhatia N, Hooper D, Nahm WK, et al. 17800 Open-label extension study evaluating the long-term safety, efficacy, and tolerability of FMX103 1.5% topical minocycline foam in the treatment of moderate to severe facial papulopustular rosacea. Journal of the American Academy of Dermatology. 2020;83(6 Supplement):AB199.

19. Hoting E, Paul E, Plewig G. Treatment of rosacea with isotretinoin. Int J Dermatol. 1986;25(10):660-3.

20. Jackson JM, Kircik LH, Lorenz DJ. Efficacy of extended-release 45 mg oral minocycline and extended-release 45 mg oral minocycline plus 15% azelaic acid in the treatment of acne rosacea. 2013;12(3):292‐8.

21. Koca R, Altinyazar HC, Ankarali H, Muhtar S, Tekin NS, Cinar S. A comparison of metronidazole 1% cream and pimecrolimus 1% cream in the treatment of patients with papulopustular rosacea: A randomized open-label clinical trial. Clin Exp Dermatol. 2010;35(3):251-6.

22. Koçak M, Yağli S, Vahapoğlu G, Ekşioğlu M. Permethrin 5% cream versus metronidazole 0.75% gel for the treatment of papulopustular rosacea. A randomized double-blind placebo-controlled study. 2002;205(3):265‐70.

23. Lee DH, Li K, Suh DH. Pimecrolimus 1% cream for the treatment of steroid-induced rosacea: an 8-week split-face clinical trial. 2008;158(5):1069‐76.

24. Maddin S. A comparison of topical azelaic acid 20% cream and topical metronidazole 0.75% cream in the treatment of patients with papulopustular rosacea. 1999;40(6 Pt 1):961‐5.

25. Miyachi Y, Yamasaki K, Fujita T, Fujii C. Metronidazole gel (0.75%) in Japanese patients with rosacea: a randomized, vehicle-controlled, phase 3 study. 2021.

26. Mostafa FF, El Harras MA, Gomaa SM, Al Mokadem S, Nassar AA, Abdel Gawad EH. Comparative study of some treatment modalities of rosacea. 2009;23(1):22‐8.

27. Mrowietz U, Kedem TH, Keynan R, Eini M, Tamarkin D, Rom D, et al. A Phase II, Randomized, Double-Blind Clinical Study Evaluating the Safety, Tolerability, and Efficacy of a Topical Minocycline Foam, FMX103, for the Treatment of Facial Papulopustular Rosacea. Am J Clin Dermatol. 2018;19(3):427-36.

28. Nct. Safety and Efficacy of Azelaic Acid Foam, 15 % in Papulopustular Rosacea. 2012.

29. Nct. Comparative Safety and Efficacy of Two Treatments in the Treatment of Inflammatory Lesions of Rosacea. 2016.

30. Nct. A Controlled Study to Assess the Efficacy, Safety and Tolerability of Oral DFD-29 Extended Release Capsules. 2017.

31. Nct. A Pilot Study on the Use of Seysara for Rosacea. 2020.

32. Sbidian E, Vicaut E, Chidiack H, Anselin E, Cribier B, Dreno B, et al. A Randomized-Controlled Trial of Oral Low-Dose Isotretinoin for Difficult-To-Treat Papulopustular Rosacea. 2016;136(6):1124‐9.

33. Schaller M, Dirschka T, Kemeny L, Briantais P, Jacovella J. Superior Efficacy with Ivermectin 1% Cream Compared to Metronidazole 0.75% Cream Contributes to a Better Quality of Life in Patients with Severe Papulopustular Rosacea: A Subanalysis of the Randomized, Investigator-Blinded ATTRACT Study. Dermatology and Therapy. 2016;6(3):427-36.

34. Solomon JA, Tyring S, Staedtler G, Sand M, Nkulikiyinka R, Shakery K. Investigator-reported efficacy of azelaic acid foam 15% in patients with papulopustular rosacea: secondary efficacy outcomes from a randomized, controlled, double-blind, phase 3 trial. Cutis. 2016;98(3):187-94.

35. Stein Gold L, Del Rosso JQ, Bhatia ND, Hooper D, Nahm W, Stuart I. Efficacy and safety of FMX103 (1.5% minocycline foam) in the treatment of moderate-to-severe papulopustular rosacea: Results from two Phase III randomized, multicenter, double-blind, vehiclecontrolled studies. J Clin Aesthet Dermatol. 2019;12(5 Supplement 1):S32.

36. Taieb A, Ortonne JP, Ruzicka T, Roszkiewicz J, Berth-Jones J, Peirone MH, et al. Superiority of ivermectin 1% cream over metronidazole 0.75% cream in treating inflammatory lesions of rosacea: A randomized, investigator-blinded trial. Br J Dermatol. 2015;172(4):1103-10.

37. Tan JKL, Girard C, Krol A, Murray HE, Papp KA, Poulin Y, et al. Randomized placebo-controlled trial of metronidazole 1% cream with sunscreen SPF 15 in treatment of rosacea. J Cutan Med Surg. 2002;6(6):529-34.

38. Thiboutot D, Thieroff-Ekerdt R, Graupe K. Efficacy and safety of azelaic acid (15%) gel as a new treatment for papulopustular rosacea: Results from two vehicle-controlled, randomized phase III studies. Journal of the American Academy of Dermatology. 2003;48(6):836-45.

39. Torok HM, Webster G, Dunlap FE, Egan N, Jarratt M, Stewart D. Combination sodium sulfacetamide 10% and sulfur 5% cream with sunscreens versus metronidazole 0.75% cream for rosacea. 2005;75(6):357‐63.

40. Tsianakas A, Pieber T, Baldwin H, Feichtner F, Alikunju S, Gautam A, et al. Minocycline Extended-Release Comparison with Doxycycline for the Treatment of Rosacea: A Randomized, Head-to-Head, Clinical Trial. The Journal of clinical and aesthetic dermatology. 2021;14(12):16-23.

41. Uslu M, Savk E, Karaman G, Sendur N. Rosacea treatment with intermediate-dose isotretinoin: Follow-up with erythema and sebum measurements. Acta Derm Venereol. 2012;92(1):73-7.

42. Webster G, Draelos ZD, Graber E, Lee MS, Dhawan S, Salman M, et al. A multicentre, randomized, double-masked, parallel group, vehicle-controlled phase IIb study to evaluate the safety and efficacy of 1% and 3% topical minocycline gel in patients with papulopustular rosacea. Br J Dermatol. 2020;183(3):471-9.

43. Wolf Jr JE, Del Rosso JQ. The CLEAR trial: Results of a large community-based study of metronidazole gel in rosacea. Cutis. 2007;79(1):73-80.
