# Supplementary material for: Efficacy of Treatments in Reducing Inflammatory Lesion Count in Rosacea: A Systematic Review
Source: J Cutan Med Surg. 2024 May 28;28(4):352–9. doi: 10.1177/12034754241253195 (PMC11408985; doi:10.1177/12034754241253195)
Supplement: sj-docx-3-cms-10.1177_12034754241253195 – Supplemental material for Efficacy of Treatments in Reducing Inflammatory Lesion Count in Rosacea: A Systematic Review [file sj-docx-3-cms-10.1177_12034754241253195.docx]

**Efficacy of treatments in reducing inflammatory lesion count in rosacea: A systematic review**

Ryan S.Q. Geng (MSc)^1^, Siddhartha Sood (BSc)^1^, Nicholas Hua (MSc)^1^, Jennifer Chen (BSc)^1^, Ronald G. Sibbald (MD)^2^, Cathryn Sibbald (MD)^3^

^1^Temerty School of Medicine, University of Toronto, Toronto, ON, Canada

^2^Dalla Lana School of Public Health & Division of Dermatology, Department of Medicine, University of Toronto, Toronto, ON, Canada

^3^Division of Pediatric Dermatology, The Hospital for Sick Children, University of Toronto, Toronto, Ontario, Canada

**Corresponding author**

Ryan Geng

1 King’s College Circle

Toronto, ON M5S 1A8

T: 647-970-9102

ryan.geng@mail.utoronto.ca

**Key words:** rosacea, inflammatory lesions, demodex

**Abstract**

**Introduction:** Rosacea is a chronic inflammatory skin condition affecting approximately 5.5% of the global population. Patients present heterogeneously with a mix of features in the central facial region, of which papules and pustules are considered to be a major feature. The identification of effective treatments for reducing inflammatory lesions in rosacea can alleviate the psychosocial burden that many rosacea patients experience, including reduced self-esteem, anxiety and social withdrawal. The objective of this systematic review is to determine the effectiveness of topical and systemic therapies in reducing lesion count in rosacea patients.

**Methods/Results:** Medline, Embase and Cochrane CENTRAL databases were searched, resulting in the inclusion of 43 clinical trials reporting on a total of 18,347 rosacea patients. The most well studied treatments include ivermectin, metronidazole, azelaic acid, minocycline and doxycycline. Oral isotretinoin was the most effective treatment in reducing inflammatory lesions and may be recommended for severe recalcitrant cases of rosacea.

**Conclusions:** Several topical and systemic therapies have demonstrated efficacy in reducing inflammatory lesion count in rosacea patients, with mechanisms of action centered around suppressing inflammation and killing *Demodex folliculorum* mites. Additional research is required to determine effective combination therapies in rosacea.

**Introduction**

Rosacea is chronic inflammatory condition of the skin that affects approximately 5.5% of the global population, with a predilection towards women.^1-3^ Rosacea presents heterogeneously with a mix of features including persistent erythema, phymatous changes, flushing, telangiectasia, papules and pustules and ocular manifestations.^4^ With the features of rosacea primarily affecting the central facial region, rosacea is associated with substantial psychosocial impact, with many rosacea patients experiencing reduced self-esteem, anxiety and social withdrawal.^5^

While the pathogenesis of rosacea remains unclear, it is likely multifactorial, involving dysregulation of the innate immune system and the neurovascular system. Several factors including high levels of cathelicidin, activation of inflammasomes and T_h_1/T_h_17 skewed response.^6^ For the formation of inflammatory lesions (ILs; papules and pustules) in rosacea, *Demodex folliculorum* mites in particular have been implicated. The role of *D. folliculorum* in rosacea pathogenesis is supported by the observation that 98.6% of rosacea patients presenting with ILs is found to have high densities of *D. folliculorum*.^7^ *D. folliculorum* mites inhabit hair follicles and have the ability to evade immune responses to establish a persistent presence. However, at higher densities, their inherent immunogenicity may overwhelm the immunosuppressive mechanisms, triggering inflammation and the appearance of ILs.^8^

Several topical and systemic treatment options are available with varying degrees of efficacy in different phenotypes. Given the psychosocial impact of the clinical features of rosacea have on patients, it is important to identify treatments that are effective in alleviating such features. The objective of this systematic review is to determine the effectiveness of topical and systemic therapies in reducing IL count in rosacea patients.

**Methods**

**Literature search**

The literature search for this systematic review (CRD42023468649) was performed according to the Preferred Reporting Items for Systematic Reviews and Meta-analyses (PRISMA) guidelines.^9^ Medline, Embase and Cochrane CENTRAL were searched from database conception to September 2023, with no exclusions on language. Databases were search with a combination of search terms including rosacea, papules, pustules and inflammatory lesions. The full search strategy is provided in the supplementary material. The removal of 133 duplicates resulted in a total of 264 studies for screening. A PRISMA flow diagram detailing the review process is provided (Supplemental Fig. S1).

**Inclusion and exclusion criteria**

All clinical trials reporting on the change in IL count post-treatment with a topical or systemic monotherapy with a treatment duration of 8-16 weeks were included. Studies that reported the change in ILs by categorical grading scales were excluded.

**Data extraction and analysis**

Articles were initially screened on basis of title and abstract, followed by full-text screening to determine final eligibility for inclusion. Relevant article references were also assessed for eligibility. NH and JC individually performed data extraction, with disagreements mediated by a third author. The change in IL count data was analyzed by calculating mean percent change in IL count and standard deviations weighted by sample size for each treatment. For studies that report on multiple time points, only the longest time point was included for analysis. The National Institute of Health quality assessment tool was used to assess methodological quality, which provides quality ratings of “good”, “fair” or “poor”. Only reports with a “good” rating were included.

**Results**

A total of 264 unique articles were identified with the outlined search strategy (Supplemental Fig. S1). Of the 43 articles included, all were RCTs with the exception of 3 uncontrolled trials, reporting on 18,347 rosacea patients (Supplemental Table 1). A table summarizing the efficacy of treatments in reducing IL counts is provided (Table 1). A diagram ranking the treatments by percent post-treatment IL reduction is also provided (Supplemental Fig. 1).

Insert Table 1

1. Adapalene [27 patients]

Adapalene was reported by 1 study in a 0.1% topical formulation applied once-daily.^10^ There was a mean 83.5% reduction in ILs among the 27 patients, representing a 1.75-fold reduction over topical placebo/vehicle.

2. Azelaic acid [1999 patients]

Azelaic acid was reported by 7 studies in either 15% or 20% topical formulations applied twice-daily.^11-17^ For the 2 studies reporting on the 20% formulation, there was a mean 74.7% reduction in ILs among the 92 patients, representing a 1.56-fold reduction over topical placebo/vehicle.^11,14^ For the 5 studies reporting on the 15% formulation, there was a mean 61.3% reduction in ILs among the 1907 patients, representing a 1.28-fold reduction over topical placebo/vehicle.^12,13,15-17^

3. Benzoyl peroxide / clindamycin [52 patients]

Benzoyl peroxide 5% / clindamycin 1% topical formulation was reported by 1 study.^18^ There was a mean 72.7% reduction in ILs among the 52 patients, representing a 1.52-fold reduction over topical placebo/vehicle.

4. Clarithromycin [20 patients]

Clarithromycin was reported by 1 study at a dose of 500mg thrice-daily.^19^ There was a mean 71.2% reduction in ILs among the 20 patients, representing a 2.58-fold reduction over systemic placebo.

5. Doxycycline [450 patients]

Doxycycline was reported by 4 studies at a dose of 40mg once-daily.^20-23^ There was a mean 49.5% reduction in ILs among the 450 patients, representing a 1.79-fold reduction over systemic placebo.

6. Isotretinoin [141 patients]

Isotretinoin was reported by 3 studies at doses ranging between of 10-50mg once-daily, with doses studied including mean 33.3mg/day (82.1% IL reduction), 20mg/day (90.9% IL reduction) and mean 15.6mg/day (92% IL reduction).^24-26^ There was a mean 87.5% reduction in ILs among the 221 patients, representing a 3.17-fold reduction over systemic placebo.

7. Ivermectin [2408 patients]

Ivermectin was reported by 6 studies in a 1% formulation applied once- or twice-daily.^27-32^ There was a mean 79% reduction in ILs among the 2408 patients, representing a 1.65-fold reduction over topical placebo/vehicle.

8. Metronidazole [2583 patients]

Metronidazole was reported by 16 studies in either 0.75% or 1% formulations applied once- or twice-daily.^10,13,14,28,31-42^ For the 13 studies reporting on the 0.75% formulation, there was a mean 70.6% reduction in ILs among the 2413 patients, representing a 1.48-fold reduction over topical placebo/vehicle.^10,13,14,28,31-33,35,36,38,39,41,42^ For the 3 studies reporting on the 1% formulation, there was a mean 67.2% reduction in ILs among the 170 patients, representing a 1.41-fold reduction over topical placebo/vehicle.^34,36,37,40^

9. Minocycline [3846 patients]

Minocycline was reported by 10 studies in either 1.5% or 3% topical formulations applied once-daily or dosed between 20-45mg once-daily.^22,23,43-50^

For the 7 studies reporting on the 1.5% formulation, there was a mean 45.9% decrease in ILs among the 3462 patients, representing a 1.28-fold reduction over topical placebo.^43-45,47-50^ For the 2 studies reporting on the 3% formulation, there was a mean 53.2% reduction in ILs among the 151 patients, representing a 1.11-fold reduction over topical placebo/vehicle.^47,50^

For the 2 studies reporting on a 20mg once-daily dose, there was a mean 53.2% reduction in ILs among the 97 patients, representing a 1.93-fold reduction over systemic placebo.^22,23^ For the 3 studies reporting on a 40-45mg once-daily dose, there was a mean 81.6% reduction in ILs among the 136 patients, representing a 2.96-fold reduction over systemic placebo.^22,23,46^

10. Permethrin [39 patients]

Permethrin was reported by 2 studies in a 5% formulation applied twice-daily.^14,38^ There was a mean 66.5% reduction in ILs among the 39 patients, representing a 1.39-fold reduction over topical placebo/vehicle.

11. Pimecrolimus [70 patients]

Pimecrolimus was reported by 3 studies in a 1% formulation applied twice-daily.^37,51,52^ There was a mean 80.3% reduction in ILs among the 70 patients, representing a 1.68-fold reduction over topical placebo/vehicle.

12. Sarecycline [72 patients]

Sarecycline was reported by 1 study at a once-daily weight-based dose according to the label.^48^ There was a mean 80% reduction in ILs among the 72 patients, representing a 2.90-fold reduction over systemic placebo.

13. Sodium sulfacetamide / sulfur [75 patients]

Sodium sulfacetamide 10% / sulfur 5% topical formulation was reported by 1 study.^41^ There was a mean 80% reduction in ILs among the 75 patients, representing a 1.67-fold reduction over topical placebo/vehicle.

**Discussion**

This review included 43 articles reporting on a total of 18,347 rosacea patients, assessing the efficacy of 13 different topical or systemic treatments in reducing IL count. The quality of evidence in this review overall was high, with all included articles being RCTs, with the exception of 3 uncontrolled trials.

Systemic treatments generally achieved greater fold-reductions in IL count over placebo compared to topical treatments. However, this is largely due to the high difference in percent IL reduction between topical placebo/vehicle and systemic placebo, 47.8% and 27.6%, respectively (Table 1). Studies assessing topical therapies often use vehicle formulations as the comparator rather than placebo. Unlike placebo, vehicle formulations function to enhance absorption and activity of drugs, and are not merely inert excipients. Rather, they often contain components that aid in skin barrier restoration and moisturization, which can soothe inflammation.^53^ These properties cannot be provided by a systemic placebo.

Amongst the systemic treatments, isotretinoin in a 10-50mg daily dosage achieved the greatest percent reductions in IL count. Isotretinoin is most commonly used in treating severe forms of acne vulgaris, with its efficacy due to its ability to inhibit comedogenesis, reduce sebum excretion and pilosebaceous unit size.^54^ While ILs in rosacea do not arise from comedones, the reduction in sebum excretion and pilosebaceous unit size may be contributory to its efficacy in rosacea. D. folliculorum are believed to play a large role in formation of ILs in rosacea. These mites inhabit hair follicles and feed on sebum and skin cells.^55^ While isotretinoin does not boast acaricidal properties, it appears to indirectly inhibit mite proliferation. The atrophy of pilosebaceous units and reduced sebum secretion induced by isotretinoin therapy may reduce the hospitability of the host skin and inhibit mite proliferation. Thus, isotretinoin is able to modify the conditions in the skin that promote demodex mite proliferation, which could explain why it is more effective than agents that simply kill the mite, such as ivermectin. In an ex vivo study, isotretinoin was found to reduce demodex mite density by 63.4%.^56^ Isotretinoin has been demonstrated to be effective in treating rosacea, particularly in cases of severe recalcitrant rosacea with papules and pustules.^26^ Isotretinoin can also inhibit neutrophil and monocyte chemotaxis, providing an anti-inflammatory effects that may also be beneficial in rosacea.^57^

Doxycycline was the most commonly studied systemic therapy, being used at a sub-antimicrobial anti-inflammatory dosage of 40mg daily. Sub-antimicrobial dosages are preferred to prevent changes to gut microbiota and reduce risk of gastrointestinal adverse reactions and development of resistance.^6^ The efficacy of doxycycline in treating rosacea is likely due to its ability to downregulate several factors involved in rosacea pathogenesis including interleukin 1β, tumour necrosis factor α (TNFα), kallikrein-5 (KLK5), cathelicidin and matrix metalloprotease 9.^58,59^

The most commonly studied topical treatments were ivermectin, metronidazole, azelaic acid and minocycline. Amongst these, ivermectin achieved the greatest percent reduction in ILs, which can be explained by its direct acaricidal and anti-inflammatory properties via inhibition of interleukin 1β and tumour necrosis factor α. However, some patients may experience an acute exacerbation of symptoms due to the sudden death of demodex mites.^6^ This transient inflammatory exacerbation is likely due to the release of immunogenic antigens from the dead mites, but may also be linked to the release of *Bacillus oleronius* bacteria that live inside the mites. *B. oleronius* is not a human cutaneous commensal organism and has been shown to trigger an inflammatory response in 73% of rosacea patients.^60^ Thus, targeting *B. oleronius* may also provide therapeutic benefit to rosacea patients. Interestingly, *B. oleronius* is susceptible to several antibiotics commonly used in rosacea, including metronidazole, doxycycline and minocycline.^60,61^ While metronidazole does not exhibit acaricidal activity towards demodex mites, it has reactive oxygen species scavenging activity, inhibits production of pro-inflammatory cytokines and exhibits bactericidal activity against *B. oleronius*.^62,63^ The efficacy of azelaic acid is due to its ability to suppress kallikrein-5 and cathelicidin production, which are part of a major inflammatory pathway contributing to rosacea pathogenesis.^64^ Topical minocycline 1.5% foam was comparable to azelaic acid 15% cream, and is believed to owe its efficacy in treating rosacea to its anti-inflammatory properties.^6^

Given that several topical and systemic therapies are effective in treating ILs in rosacea with differing mechanisms of action, combination therapies may be especially effective. Indeed, in one trial, patients treated with doxycycline 40mg and ivermectin 1% cream once-daily exhibited a mean 80.3% reduction in IL count compared to 73.6% for those treated with ivermectin monotherapy (p=0.032). Combination therapy also demonstrated a faster onset of action, with significant differences noted by week 4.^65^

Limitations of our study include a large range of patient sample sizes and heterogenous patient population. Rosacea is a chronic disease, with periods of remission and relapse. The results of this study are only applicable to the reduction of active ILs, and does not reflect remission of disease. Additional research is required to determine the effectiveness of various combination treatments in reducing IL count in rosacea.

**Conclusion**

Rosacea is a psychosocially burdensome chronic inflammatory skin condition affecting approximately 5.5% of the global population. A major feature of rosacea is inflammatory lesions, consisting of papules and pustules. Several topical and systemic therapies have demonstrated efficacy in reducing IL count in rosacea patients, with mechanisms of action centred around suppressing inflammation and killing D. folliculorum mites. The most well studied treatments for ILs in rosacea include ivermectin, metronidazole, azelaic acid, doxycycline and minocycline. Isotretinoin was found to be the most effective and could be considered for cases of severe recalcitrant rosacea.

**Acknowledgements**

None.

**Funding**

This research received no specific grant from any funding agency in the public, commercial, or not-for-profit sectors.

**Conflicting interests disclosures**

CS has received honoraria from Abbvie, Leo, Pfizer, Miravo, Novartis, UCB, Sanofi/Regeneron unrelated to this work.

RGS has received honoraria from Perfuse, Quart Medical, Novartis, Medexus Pharmaceuticals Canada along with Ontario Gov’t (Project ECHO Ontario Skin & Wound- Ministry of Health and Micro-credentials – through Ministry of Colleges and Universities and Sault College all unrelated to this work

RSQG, SS, NH and JC have no conflicts of interest to disclose.

**Data availability**

The data underlying this article are available in the article and in its online supplementary material.

**Ethics**

An ethics statement is not applicable because this study is based exclusively on published literature.

**References**

1. Gether L, Overgaard LK, Egeberg A, Thyssen JP. Incidence and prevalence of rosacea: a systematic review and meta-analysis. *Br J Dermatol*. Aug 2018;179(2):282-289. doi:10.1111/bjd.16481

2. Abram K, Silm H, Maaroos HI, Oona M. Risk factors associated with rosacea. *J Eur Acad Dermatol Venereol*. May 2010;24(5):565-71. doi:10.1111/j.1468-3083.2009.03472.x

3. Tan J, Berg M. Rosacea: current state of epidemiology. *J Am Acad Dermatol*. Dec 2013;69(6 Suppl 1):S27-35. doi:10.1016/j.jaad.2013.04.043

4. Tan J, Almeida LM, Bewley A, et al. Updating the diagnosis, classification and assessment of rosacea: recommendations from the global ROSacea COnsensus (ROSCO) panel. *Br J Dermatol*. Feb 2017;176(2):431-438. doi:10.1111/bjd.15122

5. Huynh TT. Burden of Disease: The Psychosocial Impact of Rosacea on a Patient's Quality of Life. *Am Health Drug Benefits*. Jul 2013;6(6):348-54.

6. Chen C, Wang P, Zhang L, et al. Exploring the Pathogenesis and Mechanism-Targeted Treatments of Rosacea: Previous Understanding and Updates. *Biomedicines*. 2023;11(8). doi:10.3390/biomedicines11082153

7. Forton FM, De Maertelaer V. Two Consecutive Standardized Skin Surface Biopsies: An Improved Sampling Method to Evaluate Demodex Density as a Diagnostic Tool for Rosacea and Demodicosis. *Acta Derm Venereol*. Feb 8 2017;97(2):242-248. doi:10.2340/00015555-2528

8. Forton FMN. Rosacea, an infectious disease: why rosacea with papulopustules should be considered a demodicosis. A narrative review. *J Eur Acad Dermatol Venereol*. Jul 2022;36(7):987-1002. doi:10.1111/jdv.18049

9. Page MJ, McKenzie JE, Bossuyt PM, et al. The PRISMA 2020 statement: an updated guideline for reporting systematic reviews. *BMJ*. Mar 29 2021;372:n71. doi:10.1136/bmj.n71

10. Altinyazar HC, Koca R, Tekin NS, Eştürk E. Adapalene vs. metronidazole gel for the treatment of rosacea. 2005;44(3):252‐255. doi:10.1111/j.1365-4632.2004.02130.x

11. Bjerke R, Fyrand O, Graupe K. Double-blind comparison of azelaic acid 20% cream and its vehicle in treatment of papulo-pustular rosacea. 1999;79(6):456‐459. doi:10.1080/000155599750009906

12. Draelos ZD, Elewski BE, Harper JC, et al. Randomized, phase III, double-blind, vehicle-controlled clinical trial to evaluate the safety and efficacy of 12 weeks of twice-daily azelaic acid foam, 15% in papulopustular rosacea. *Journal of the American Academy of Dermatology*. 2015;72(5 SUPPL. 1):AB59.

13. Elewski BE, Fleischer Jr AB, Pariser DM. A Comparison of 15% Azelaic Acid Gel and 0.75% Metronidazole Gel in the Topical Treatment of Papulopustular Rosacea: Results of a Randomized Trial. *Arch Dermatol*. 2003;139(11):1444-1450. doi:<https://dx.doi.org/10.1001/archderm.139.11.1444>

14. Mostafa FF, El Harras MA, Gomaa SM, Al Mokadem S, Nassar AA, Abdel Gawad EH. Comparative study of some treatment modalities of rosacea. 2009;23(1):22‐28. doi:10.1111/j.1468-3083.2008.02940.x

15. Nct. Safety and Efficacy of Azelaic Acid Foam, 15 % in Papulopustular Rosacea. 2012;

16. Solomon JA, Tyring S, Staedtler G, Sand M, Nkulikiyinka R, Shakery K. Investigator-reported efficacy of azelaic acid foam 15% in patients with papulopustular rosacea: secondary efficacy outcomes from a randomized, controlled, double-blind, phase 3 trial. *Cutis*. 2016;98(3):187-194.

17. Thiboutot D, Thieroff-Ekerdt R, Graupe K. Efficacy and safety of azelaic acid (15%) gel as a new treatment for papulopustular rosacea: Results from two vehicle-controlled, randomized phase III studies. *Journal of the American Academy of Dermatology*. 2003;48(6):836-845. doi:<https://dx.doi.org/10.1067/mjd.2003.308>

18. Breneman D, Savin R, VandePol C, Vamvakias G, Levy S, Leyden J. Double-blind, randomized, vehicle-controlled clinical trial of once-daily benzoyl peroxide/clindamycin topical gel in the treatment of patients with moderate to severe rosacea. 2004;43(5):381‐387. doi:10.1111/j.1365-4632.2004.02283.x

19. Bamford JT, Tilden RL, Blankush JL, Gangeness DE. Effect of treatment of Helicobacter pylori infection on rosacea. 1999;135(6):659‐663. doi:10.1001/archderm.135.6.659

20. Del Rosso JQ, Webster GF, Jackson M, et al. Two randomized phase III clinical trials evaluating anti-inflammatory dose doxycycline (40-mg doxycycline, USP capsules) administered once daily for treatment of rosacea. *Journal of the American Academy of Dermatology*. 2007;56(5):791-802. doi:<https://dx.doi.org/10.1016/j.jaad.2006.11.021>

21. Di Nardo A, Holmes AD, Muto Y, et al. Improved clinical outcome and biomarkers in adults with papulopustular rosacea treated with doxycycline modified-release capsules in a randomized trial. 2016;74(6):1086‐1092. doi:10.1016/j.jaad.2016.01.023

22. Nct. A Controlled Study to Assess the Efficacy, Safety and Tolerability of Oral DFD-29 Extended Release Capsules. 2017;

23. Tsianakas A, Pieber T, Baldwin H, et al. Minocycline Extended-Release Comparison with Doxycycline for the Treatment of Rosacea: A Randomized, Head-to-Head, Clinical Trial. *The Journal of clinical and aesthetic dermatology*. 2021;14(12):16-23.

24. Sbidian E, Vicaut E, Chidiack H, et al. A Randomized-Controlled Trial of Oral Low-Dose Isotretinoin for Difficult-To-Treat Papulopustular Rosacea. 2016;136(6):1124‐1129. doi:10.1016/j.jid.2016.01.025

25. Uslu M, Savk E, Karaman G, Sendur N. Rosacea treatment with intermediate-dose isotretinoin: Follow-up with erythema and sebum measurements. *Acta Derm Venereol*. 2012;92(1):73-77. doi:<https://dx.doi.org/10.2340/00015555-1204>

26. Hoting E, Paul E, Plewig G. Treatment of rosacea with isotretinoin. *Int J Dermatol*. Dec 1986;25(10):660-3. doi:10.1111/j.1365-4362.1986.tb04533.x

27. Euctr HU. A DOUBLE BLIND, VEHICLE CONTROLLED, PARALLEL GROUP STUDY ASSESSING THE ACTIVITY OF CD5024 1% CREAM IN SUBJECTS WITH PAPULOPUSTULAR ROSACEA OVER 12 WEEKS TREATMENT. 2010;

28. Foley P, Taieb A, Ruzicka T, Peirone MH, Jacovella J. Comparative efficacy and safety of ivermectin 1% cream and metronidazole 0.75% cream in the novel treatment of papulopustular rosacea: The ATTRACT (assessment of a topical treatment in rosacea - activity, compliance, tolerability) study. *Australas J Dermatol*. 2016;57(SUPPL. 1):33. doi:<https://dx.doi.org/10.1111/ajd.12480>

29. Gold LS, Kircik L, Fowler J, et al. Efficacy and safety of ivermectin 1% cream in treatment of papulopustular rosacea: Results of two randomized, double-blind, vehicle-controlled pivotal studies. *Journal of Drugs in Dermatology*. 2014;13(3):316-323.

30. Nct. Comparative Safety and Efficacy of Two Treatments in the Treatment of Inflammatory Lesions of Rosacea. 2016;

31. Schaller M, Dirschka T, Kemeny L, Briantais P, Jacovella J. Superior Efficacy with Ivermectin 1% Cream Compared to Metronidazole 0.75% Cream Contributes to a Better Quality of Life in Patients with Severe Papulopustular Rosacea: A Subanalysis of the Randomized, Investigator-Blinded ATTRACT Study. *Dermatology and Therapy*. 2016;6(3):427-436. doi:<https://dx.doi.org/10.1007/s13555-016-0133-6>

32. Taieb A, Ortonne JP, Ruzicka T, et al. Superiority of ivermectin 1% cream over metronidazole 0.75% cream in treating inflammatory lesions of rosacea: A randomized, investigator-blinded trial. *Br J Dermatol*. 2015;172(4):1103-1110. doi:<https://dx.doi.org/10.1111/bjd.13408>

33. Aronson IK, Rumsfield JA, West DP, Alexander J, Fischer JH, Paloucek FP. Evaluation of topical metronidazole gel in acne rosacea. 1987;21(4):346‐351. doi:10.1177/106002808702100410

34. Bitar A, Bourgouin J, Dore N, et al. A double-blind randomised study of metronidazole (Flagyl®) 1% cream in the treatment of acne rosacea. A placebo-controlled study. 1990;2(4):242‐248.

35. Bleicher PA, Charles JH, Sober AJ. Topical metronidazole therapy for rosacea. 1987;123(5):609‐614.

36. Dahl MV, Jarratt M, Kaplan D, Tuley MR, Baker MD. Once-daily topical metronidazole cream formulations in the treatment of the papules and pustules of rosacea. 2001;45(5):723‐730. doi:10.1067/mjd.2001.116219

37. Koca R, Altinyazar HC, Ankarali H, Muhtar S, Tekin NS, Cinar S. A comparison of metronidazole 1% cream and pimecrolimus 1% cream in the treatment of patients with papulopustular rosacea: A randomized open-label clinical trial. *Clin Exp Dermatol*. 2010;35(3):251-256. doi:<https://dx.doi.org/10.1111/j.1365-2230.2009.03427.x>

38. Koçak M, Yağli S, Vahapoğlu G, Ekşioğlu M. Permethrin 5% cream versus metronidazole 0.75% gel for the treatment of papulopustular rosacea. A randomized double-blind placebo-controlled study. 2002;205(3):265‐270. doi:10.1159/000065849

39. Miyachi Y, Yamasaki K, Fujita T, Fujii C. Metronidazole gel (0.75%) in Japanese patients with rosacea: a randomized, vehicle-controlled, phase 3 study. 2021;doi:10.1111/1346-8138.16254

40. Tan JKL, Girard C, Krol A, et al. Randomized placebo-controlled trial of metronidazole 1% cream with sunscreen SPF 15 in treatment of rosacea. *J Cutan Med Surg*. 2002;6(6):529-534. doi:<https://dx.doi.org/10.1007/s10227-001-0144-4>

41. Torok HM, Webster G, Dunlap FE, Egan N, Jarratt M, Stewart D. Combination sodium sulfacetamide 10% and sulfur 5% cream with sunscreens versus metronidazole 0.75% cream for rosacea. 2005;75(6):357‐363.

42. Wolf Jr JE, Del Rosso JQ. The CLEAR trial: Results of a large community-based study of metronidazole gel in rosacea. *Cutis*. 2007;79(1):73-80.

43. Del Rosso JQ, Stein Gold L, Kircik L, et al. Integrated safety and efficacy analysis of FMX103 1.5% topical minocycline foam for the treatment of moderate-to-severe papulopustular rosacea: results from two Phase III studies. 2021;14(5 SUPPL 1):S30.

44. Gold LS, Del Rosso JQ, Kircik L, et al. Minocycline 1.5% foam for the topical treatment of moderate to severe papulopustular rosacea: results of 2 phase 3, randomized, clinical trials. 2020;82(5):1166‐1173. doi:10.1016/j.jaad.2020.01.043

45. Gold LS, Del Rosso JQ, Kircik L, et al. 17800 Open-label extension study evaluating the long-term safety, efficacy, and tolerability of FMX103 1.5% topical minocycline foam in the treatment of moderate to severe facial papulopustular rosacea. *Journal of the American Academy of Dermatology*. 2020;83(6 Supplement):AB199. doi:<https://dx.doi.org/10.1016/j.jaad.2020.06.886>

46. Jackson JM, Kircik LH, Lorenz DJ. Efficacy of extended-release 45 mg oral minocycline and extended-release 45 mg oral minocycline plus 15% azelaic acid in the treatment of acne rosacea. 2013;12(3):292‐298.

47. Mrowietz U, Kedem TH, Keynan R, et al. A Phase II, Randomized, Double-Blind Clinical Study Evaluating the Safety, Tolerability, and Efficacy of a Topical Minocycline Foam, FMX103, for the Treatment of Facial Papulopustular Rosacea. *Am J Clin Dermatol*. 2018;19(3):427-436. doi:<https://dx.doi.org/10.1007/s40257-017-0339-0>

48. Nct. A Pilot Study on the Use of Seysara for Rosacea. 2020;

49. Stein Gold L, Del Rosso JQ, Bhatia ND, Hooper D, Nahm W, Stuart I. Efficacy and safety of FMX103 (1.5% minocycline foam) in the treatment of moderate-to-severe papulopustular rosacea: Results from two Phase III randomized, multicenter, double-blind, vehiclecontrolled studies. *J Clin Aesthet Dermatol*. 2019;12(5 Supplement 1):S32.

50. Webster G, Draelos ZD, Graber E, et al. A multicentre, randomized, double-masked, parallel group, vehicle-controlled phase IIb study to evaluate the safety and efficacy of 1% and 3% topical minocycline gel in patients with papulopustular rosacea. *Br J Dermatol*. 2020;183(3):471-479. doi:<https://dx.doi.org/10.1111/bjd.18857>

51. Lee DH, Li K, Suh DH. Pimecrolimus 1% cream for the treatment of steroid-induced rosacea: an 8-week split-face clinical trial. 2008;158(5):1069‐1076. doi:10.1111/j.1365-2133.2008.08496.x

52. Maddin S. A comparison of topical azelaic acid 20% cream and topical metronidazole 0.75% cream in the treatment of patients with papulopustular rosacea. 1999;40(6 Pt 1):961‐965. doi:10.1016/s0190-9622(99)70085-x

53. Danby SG, Draelos ZD, Gold LFS, et al. Vehicles for atopic dermatitis therapies: more than just a placebo. *Journal of Dermatological Treatment*. 2022/02/17 2022;33(2):685-698. doi:10.1080/09546634.2020.1789050

54. Layton A. The use of isotretinoin in acne. *Dermatoendocrinol*. May 2009;1(3):162-9. doi:10.4161/derm.1.3.9364

55. Rather PA, Hassan I. Human demodex mite: the versatile mite of dermatological importance. *Indian J Dermatol*. Jan 2014;59(1):60-6. doi:10.4103/0019-5154.123498

56. Clanner-Engelshofen BM, Stander LM, Steegmuller T, et al. First ex vivo cultivation of human Demodex mites and evaluation of different drugs on mite proliferation. *J Eur Acad Dermatol Venereol*. Dec 2022;36(12):2499-2503. doi:10.1111/jdv.18468

57. Norris DA, Osborn R, Robinson W, Tonnesen MG. Isotretinoin Produces Significant Inhibition of Monocyte and Neutrophil Chemotaxis In Vivo in Patients With Cystic Acne. *J Invest Dermatol*. 1987/07/01/ 1987;89(1):38-43. doi:<https://doi.org/10.1111/1523-1747.ep12580370>

58. Kanada KN, Nakatsuji T, Gallo RL. Doxycycline indirectly inhibits proteolytic activation of tryptic kallikrein-related peptidases and activation of cathelicidin. *J Invest Dermatol*. May 2012;132(5):1435-42. doi:10.1038/jid.2012.14

59. Stechmiller J, Cowan L, Schultz G. The role of doxycycline as a matrix metalloproteinase inhibitor for the treatment of chronic wounds. *Biol Res Nurs*. Apr 2010;11(4):336-44. doi:10.1177/1099800409346333

60. Lacey N, Delaney S, Kavanagh K, Powell FC. Mite-related bacterial antigens stimulate inflammatory cells in rosacea. *Br J Dermatol*. Sep 2007;157(3):474-81. doi:10.1111/j.1365-2133.2007.08028.x

61. Ávila MY, Martínez-Pulgarín DF, Rizo Madrid C. Topical ivermectin-metronidazole gel therapy in the treatment of blepharitis caused by Demodex spp.: A randomized clinical trial. *Contact Lens and Anterior Eye*. 2021/06/01/ 2021;44(3):101326. doi:<https://doi.org/10.1016/j.clae.2020.04.011>

62. Forton F, Seys B, Marchal JL, Song AM. Demodex folliculorum and topical treatment: acaricidal action evaluated by standardized skin surface biopsy. *Br J Dermatol*. Mar 1998;138(3):461-6. doi:10.1046/j.1365-2133.1998.02125.x

63. Narayanan S, Hunerbein A, Getie M, Jackel A, Neubert RH. Scavenging properties of metronidazole on free oxygen radicals in a skin lipid model system. *J Pharm Pharmacol*. Aug 2007;59(8):1125-30. doi:10.1211/jpp.59.8.0010

64. Coda AB, Hata T, Miller J, et al. Cathelicidin, kallikrein 5, and serine protease activity is inhibited during treatment of rosacea with azelaic acid 15% gel. *J Am Acad Dermatol*. Oct 2013;69(4):570-7. doi:10.1016/j.jaad.2013.05.019

65. Schaller M, Kemeny L, Havlickova B, et al. A randomized phase 3b/4 study to evaluate concomitant use of topical ivermectin 1% cream and doxycycline 40-mg modified-release capsules, versus topical ivermectin 1% cream and placebo in the treatment of severe rosacea. *J Am Acad Dermatol*. Feb 2020;82(2):336-343. doi:10.1016/j.jaad.2019.05.063

**Table**

**Table 1.** Efficacy of treatment regimens in reducing IL count in rosacea patients.

| **Treatment regimen** | **N studies** | **N patients** | **Mean % reduction in ILs** | **Fold reduction over placebo** |
| --- | --- | --- | --- | --- |
| **Topical therapies** | | | | |
| Adapalene 0.1% QD | 1 | 27 | 83.5 | 1.75 |
| Pimecrolimus 1% BID | 3 | 70 | 80.3 | 1.68 |
| SS 10% / sulfur 5% BID | 1 | 75 | 80 | 1.67 |
| Ivermectin 1% QD-BID | 6 | 2408 | 79 | 1.65 |
| Azelaic acid 20% BID | 2 | 92 | 74.7 | 1.56 |
| BPO 5% / clindamycin 1% QD | 1 | 52 | 72.7 | 1.52 |
| Metronidazole 0.75% QD-BID | 13 | 2413 | 70.6 | 1.48 |
| Metronidazole 1% QD-BID | 3 | 170 | 67.2 | 1.41 |
| Permethrin 5% BID | 2 | 39 | 66.5 | 1.39 |
| Azelaic acid 15% BID | 5 | 1907 | 61.3 | 1.28 |
| Minocycline 1.5% QD | 7 | 3462 | 61.3 | 1.28 |
| Minocycline 3% QD | 2 | 151 | 53.2 | 1.11 |
| Placebo/Vehicle QD-BID | 23 | 5956 | 47.8 | - |
| **Systemic therapies** | | | | |
| Isotretinoin 10-50mg QD | 3 | 221 | 87.5 | 3.17 |
| Minocycline 40-45mg QD | 3 | 136 | 81.6 | 2.96 |
| Sarecycline weight-dosed QD | 1 | 72 | 80 | 2.90 |
| Clarithromycin 500mg TID | 1 | 20 | 71.2 | 2.58 |
| Minocycline 20mg QD | 2 | 97 | 53.2 | 1.93 |
| Doxycycline 40mg QD | 4 | 450 | 49.5 | 1.79 |
| Systemic placebo QD-TID | 6 | 529 | 27.6 | - |

BPO = benzoyl peroxide, SS = sodium sulfacetamide

**Figure legend**

**Figure 1.** Efficacy of treatment regimens in reducing IL count in rosacea patients. The mean percent reduction in ILs is represented by the bullet for each treatment. Bars represent standard deviations. Abbreviations: BPO = benzoyl peroxide, SS = sodium sulfacetamide.
